# Impact on an Urgent Care Clinic of a New Freestanding Emergency Department in a Resource-Scarce Area

**DOI:** 10.31486/toj.21.0146

**Published:** 2022

**Authors:** Diana Hamer, Glenn N. Jones, Michael R. Loewe, Mandi W. Musso

**Affiliations:** ^1^ Louisiana State University Health Sciences Center, School of Medicine, Baton Rouge Campus, Baton Rouge, LA; ^2^ Division of Academic Affairs, Our Lady of the Lake Regional Medical Center, Baton Rouge, LA

**Keywords:** *Emergency department*, *healthcare disparities*, *healthcare utilization*, *urgent care*

## Abstract

**Background:** Convenience clinics—such as urgent care centers (UCCs), retail clinics, and freestanding emergency departments (FSEDs)—where patients can receive treatment for a variety of medical conditions have increased in number and popularity. We quantify the impact an FSED had on UCC visits in an underserved area in North Baton Rouge, Louisiana.

**Methods:** All FSED and UCC visits were abstracted from 2015 to 2020. Visits were classified using *International Classification of Diseases, Tenth Revision* codes. We used a time series analysis to evaluate the association of nonemergent and emergent visits to the UCC after the opening of the FSED. Visits were also aggregated at the census block group (neighborhood) level. Demographic characteristics and the neighborhood Area Deprivation Index were used to compare UCC utilization before and after the FSED opened and to describe the visits to the UCC and the FSED.

**Results:** We found a difference in the demographic composition of patients presenting to the UCC after the FSED opened. Emergent visits decreased at the UCC, but nonemergent visits did not change after the FSED opened. The majority of visits to the FSED were nonemergent, and the proportion of nonemergent visits to the FSED increased during the hours that the UCC was closed. The majority of visits to the FSED came from neighborhoods with a high Area Deprivation Index.

**Conclusion:** The opening of an FSED resulted in a reduction of emergent visits to the UCC without impacting the number of nonemergent visits. The opening of an FSED in a poor, healthcare-resource-scarce area resulted in significantly more patients from deprived neighborhoods being treated at the FSED and UCC.

## INTRODUCTION

The number and types of convenience clinics as alternatives to hospital-based emergency departments (EDs) for the treatment of a variety of medical conditions are increasing. Urgent care centers (UCCs), retail clinics, and freestanding emergency departments (FSEDs) that accept walk-in appointments and involve shorter wait times than hospital-based EDs are growing in popularity.^1^

FSEDs provide services similar to the services provided by hospital-based EDs, including imaging and laboratory services. One potential benefit of FSEDs is improving access to care for patients who are not located near hospital-based EDs.^2,3^ However, independent FSEDs, in contrast to hospital-affiliated FSEDs, are not bound by federal regulations, such as the Emergency Medical Treatment and Labor Act (EMTALA), to provide care for all patients, and regulatory oversight is determined by individual states.^4^ Consequently, the impact of FSEDs is not equal for all demographics.^5^ Data indicate that FSEDs tend to treat younger, privately insured patients with less severe illnesses compared to hospital-based EDs.^6^ Further, FSEDs have traditionally tended to locate further from public transit and therefore have catered to affluent populations with options for care other than hospital-based EDs.^3,7-9^

UCCs treat the majority of the most common diagnoses seen at FSEDs.^1^ With regard to cost, FSEDs and hospital-based EDs have higher out-of-pocket liabilities for patients compared to UCCs.^1^ On the other hand, UCCs do not always accept Medicaid, and many require a copay before a patient is seen by a medical provider, regardless of insurance status.^10^

In 2013, the state-run hospital closed in North Baton Rouge, Louisiana, and the remaining hospital-based ED closed in 2015, leaving no access to emergency medical care in an area within a 10-mile radius.^11^ In response to the 2013 hospital closure, the Franciscan Missionaries of Our Lady (FMOL) Health System opened a UCC in this area in 2013.^12^ On November 15, 2017, the FMOL Health System opened an FSED affiliated with Our Lady of the Lake Regional Medical Center in this predominantly minority neighborhood where 33.4% of the residents live below the poverty level.^13^ The FSED opened in the same building as the UCC and offered a triage system for patients who presented for care. Under Louisiana requirements, FSEDs are required to be affiliated with an existing licensed hospital, must be open 24 hours per day, 7 days per week, and are required to receive ambulances.^4^

The purpose of this study was to quantify the impact the FSED had on UCC visits in this underserved area in North Baton Rouge, Louisiana.

## METHODS

### Study Design and Setting

We examined visits to the FMOL-affiliated North Baton Rouge UCC from October 1, 2015, until March 1, 2020. We examined visits from the FSED facility from its opening on November 15, 2017, until March 1, 2020. We chose this end date because of changes in healthcare utilization resulting from the state-mandated stay-at-home order at the beginning of the coronavirus disease 2019 pandemic.^14,15^ Of note, all FMOL-affiliated facilities accept Medicaid. All visits by adult patients ≥18 years were included in this analysis.

The UCC is open from 7 AM until 12 AM, 7 days per week. The UCC does not schedule appointments. The FSED is open 24 hours per day, 7 days per week. All patients present to a main entrance and undergo triage and medical screening processes during the hours of UCC operation. If the patient's presentation is determined to be nonurgent, the patient is provided with the option to be seen at the UCC or to follow up with a primary care physician (PCP). Urgent complaints are triaged to the FSED. When the UCC is closed, all patients are routed to the FSED without triage. Patients who presented to the FSED and were judged to require admission to the hospital were transported to a hospital-based ED where they were evaluated and admitted if necessary.

This study was approved by the Louisiana State University Health Sciences Center Institutional Review Board.

### Methods and Measurement

#### Visit Variables

The following data were abstracted from the electronic medical record: age, biological sex, insurance status, emergency severity index (ESI), length of stay, admission status, and address. Patients’ diagnoses were based on the *International Classification of Diseases, Tenth Revision* codes. FSED visits and UCC visits were classified using the New York University ED profiling algorithm.^16-18^ In the original work by Billings and colleagues, ED visits were coded as “ED Care Needed: Not Preventable,” “ED Care Needed: Preventable/Avoidable,” “Emergent: PCP Treatable,” “Non-Emergent,” “Alcohol/drug,” “Injury,” “Psychiatric,” and “Unclassified.”^16^ However, we used the method validated by Ballard et al, combining “ED Care Needed: Not Preventable” and “ED Care Needed: Preventable/Avoidable” into an “Emergent” category and “PCP Treatable” and “Non-Emergent” into a “Non-Emergent” category.^19^ Furthermore, visits to the FSED were classified as occurring during the UCC operating hours (7 AM until 12 AM) or when the UCC was closed (12 AM until 7 AM) and whether the visit resulted in a hospital admission or ED discharge.

#### Data Analysis

Statistical analyses were computed using IBM SPSS Statistics, version 26 (IBM Corporation). Visit demographic characteristics and classification were compared using Fisher exact test or chi-square test for nonparametric variables. All continuous variables were compared with the Mann-Whitney *U* test. Categorical demographic characteristics and visit classification are expressed as count and proportion, and continuous variables are reported as median and interquartile range (IQR).

We conducted a time series analysis using an autoregression AR(1) model to evaluate the association of nonemergent and emergent visits classified by the Billings algorithm to the UCC before (October 1, 2015, to November 15, 2017) and after (November 16, 2017, to March 1, 2020) the opening of the FSED.

#### Census Block Group Variables

We transformed the patient address associated with each visit into latitude and longitude coordinates using Texas A&M University GeoServices^20^ and aggregated UCC and FSED visits to the census block group level (ie, neighborhood) using ArcGIS Desktop Software, version 10.6 (Esri).^21^ The census block group is the smallest geographic area for which the US Census Bureau publishes decennial census data.^22^ Louisiana consists of 64 parishes that are divided into 3,471 census block groups.^23^ Census block groups were classified into quintiles using the 2015 and 2019 Area Deprivation Index (ADI) for the study period prior to (October 2015 to November 2017) and after (November 2017 to March 2020) the FSED opened. The ADI is a composite index of community socioeconomic measures scored from 1 to 100, with 1 being the least socioeconomically deprived block group and 100 being the most deprived.^24^ Studies have demonstrated an association between the neighborhood deprivation level and all-cause mortality, as well as all-cause 30-day hospital readmission.^25^ We calculated the total adult (≥18 years) noninstitutionalized population using the 2016 American Community Survey 5-Year Estimates and the 2019 American Community Survey 5-Year Estimates to obtain the UCC utilization per 1,000 inhabitants prior to and after the opening of the FSED.^26^ The UCC utilization before and after the FSED opened was compared with Fisher exact test using the number of visits from the census block group divided by the census block group's total adult population. ADI quintiles utilization is expressed as number of visits per 1,000 people.

## RESULTS

### Characteristics of Study Population

This study examined data from 136,093 UCC visits and 26,458 FSED visits. Table 1 shows the characteristics of patients presenting to the UCC before and after the opening of the FSED and the characteristics of patients with FSED visits. Most notably, the median (IQR) age of patients presenting to the UCC decreased from 36.1 years (26.5-50.5 years) to 35.1 years (25.9-49.8 years, *P*<0.001). The racial/ethnic composition of patients presenting to the UCC significantly changed (χ*^2^*=255.36, *P*<0.001) after the opening of the FSED. The UCC served a higher percentage of Black patients after the FSED opened. Similarly, the insurance composition changed; significantly more patients covered by Medicaid presented to the UCC (χ^2^=7,470.95, *P*<0.001) in the months following the opening of the FSED. Only 12.8% of patients who presented to the FSED were admitted to the hospital-based ED.

**Table 1.** Demographic Characteristics of Patients Presenting to the Urgent Care Clinic and Freestanding Emergency Department

	Urgent Care Clinic			
Variable	Before^a^	After^a^	*P* Value	Freestanding Emergency Department
Number of visits	63,483	72,610		26,458
Age, years, median (IQR)^b^	36.1 (26.5-50.5)	35.1 (25.9-49.8)	<0.001	41.05 (29.3-56.2)
Race/ethnicity^c^				
White/Caucasian	5,126 (8.1)	4,502 (6.2)	<0.001	1,951 (7.4)
Black/African American	56,479 (89.0)	66,446 (91.5)		23,781 (89.9)
Hispanic	648 (1.0)	530 (0.7)		285 (1.1)
Asian	202 (0.3)	163 (0.2)		60 (0.2)
Other	1,028 (1.6)	969 (1.3)		381 (1.4)
Sex^c^^,^^d^				
Female	38,916 (61.3)	48,242 (66.4)	<0.001	15,355 (58.0)
Male	24,563 (38.7)	24,363 (33.6)		11,103 (42.0)
Insurance status^c^				
Private	9,186 (14.5)	9,414 (13)	<0.001	3,497 (13.2)
Medicaid	25,533 (40.2)	41,559 (57.2)		13,771 (52.0)
Medicare	5,204 (8.2)	8,920 (12.3)		5,245 (19.8)
Self-pay	23,560 (37.1)	12,717 (17.5)		3,945 (14.9)
Emergency department to hospital admission				3,387 (12.8)

^a^Before, 26 months before the opening of the freestanding emergency department (October 1, 2015, to November 15, 2017); After, 28 months after the opening of the freestanding emergency department (November 16, 2017, to March 1, 2020).

^b^Mann-Whitney *U* test comparing the independent distribution of nonparametrically distributed data.

^c^Chi-square test of independence.

^d^Total n for the Before column is 63,479. Total n for the After column is 72,605.

Notes: Data are presented as n (%) unless otherwise indicated. *P*<0.05 indicates statistical significance.

IQR, interquartile range.

**Figure.**
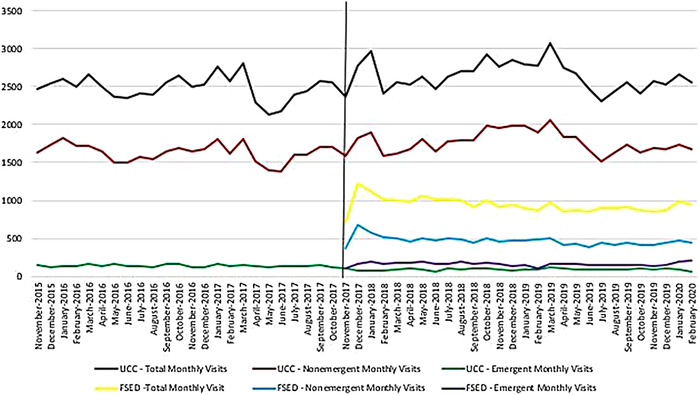
Monthly visits to the urgent care clinic (UCC) and freestanding emergency department (FSED) by nonemergent and emergent classification.

### Time Series Analysis

Prior to the opening of the FSED on November 15, 2017, the UCC had 2,441.65 visits per month (Table 2, Figure). After the FSED opened, the average decrease in UCC visits was 120.88 visits per month, but this difference was not statistically significant (*t*=1.704, *P*=0.095). Emergent visits decreased from a mean of 138.11 to a mean of 88.71 visits per month after the opening of the FSED, and the AR(1) model showed that this decrease was significant (estimate=–48.37, SE=3.7, *t*=–13.059, *P*<0.001). The mean number of nonemergent visits per month in the UCC increased after the FSED opened, but the change was not significant (estimate=91.81, SE=60.34, *t*=1.521, *P*=0.134).

**Table 2.** Mean Visits per Month, Emergency Department Outcomes, and AR(1) Model Estimations for Urgent Care Clinic Visits

Visit Classification/Outcome	Before,^a^ Mean Visits per Month	After,^a^ Mean Visits per Month	Estimate^b^	Standard Error	*t* Test	*P* Value
Urgent care clinic visits						
Total	2,441.65	1,910.78	–120.88	70.95	1.704	0.095
Nonemergent	1,603.38	1,739.32	91.81	60.34	1.521	0.134
Emergent	138.11	88.71	–48.37	3.70	–13.059	<0.001
Freestanding emergency department visits						
Total		944.92				
Nonemergent		477.07				
Emergent		163.42				
Freestanding emergency department outcomes						
Discharge		823.96				
Hospital admission		120.96				

^a^Before, 26 months before the opening of the freestanding emergency department (October 1, 2015, to November 15, 2017); After, 28 months after the opening of the freestanding emergency department (November 16, 2017, to March 1, 2020).

^b^Difference between the mean before the freestanding emergency department opening and the mean after the freestanding emergency department opening after adjusting for autocorrelation. A negative estimate indicates a decrease in visits between the 2 time periods, while a positive estimate indicates an increase.

Note: *P*<0.05 indicates statistical significance.

We examined the FSED visit composition when the UCC was open vs when the UCC was closed (Table 3). Because 3,013 patients did not have a documented arrival time, their visits could not be included in the open vs closed analysis (n=23,445). Nineteen additional patients did not have a documented ESI, so these patients were not included in the analysis (n=23,426). During UCC open hours, 46.8% of visits to the FSED were classified as nonemergent compared to 55.6% when the UCC was closed (χ*^2^*=50.902, *P*<0.001). Conversely, emergent visits decreased from 19.1% during UCC open hours to 11.4% when the UCC was closed (χ*^2^*=154.098, *P*<0.001). Alcohol/drug- and psychiatric-related visits were less frequently observed during the hours when the UCC was closed, but the proportion of visits classified as injury significantly increased when the UCC was closed. The analysis also showed a significant change in the ESI classification of FSED visits during UCC closed hours (χ*^2^*=5,398.068, *P*<0.001), with the majority of visits classified as ESI 2 and 3 (less urgent and urgent, respectively).

**Table 3.** Classification and Severity of Visits to the Freestanding Emergency Department by Urgent Care Clinic Status

	Urgent Care Clinic		
Visit Classification/Severity	Open^a^	Closed^a^	*P* Value^b^
Classification			
Nonemergent	7,404 (46.8)	4,227 (55.6)	<0.001
Emergent	3,030 (19.1)	869 (11.4)	<0.001
Alcohol/drug	251 (1.6)	71 (0.9)	<0.001
Psychiatric	228 (1.4)	84 (1.1)	0.040
Injury	1,784 (11.3)	1,109 (14.6)	<0.001
Unclassified	3,139 (19.8)	1,249 (16.4)	<0.001
Emergency severity index			
Immediate – 5	79 (0.5)	27 (0.4)	<0.001
Emergent – 4	2,218 (14.0)	431 (5.7)	
Urgent – 3	12,393 (78.3)	3,476 (45.7)	
Less urgent – 2	939 (5.9)	3,299 (43.4)	
Nonurgent – 1	191 (1.2)	373 (4.9)	

^a^Open, 7:00 AM to 12:00 AM; Closed, 12:00 AM to 7:00 AM.

^b^Chi-square test of independence for difference between visits during urgent care clinic open and closed hours by individual visit classification and overall emergency severity index.

Notes: Because 3,013 patients did not have a documented arrival time, their visits could not be included in the open vs closed analysis (n=23,445). Nineteen additional patients did not have a documented emergency severity index, so these patients were not included in the analysis (n=23,426). Data are presented as n (%). *P*<0.05 indicates statistical significance.

### Area Deprivation Index

We calculated the UCC visits per 1,000 people for 549 census block groups of the UCC catchment area (Table 4). In 2015, 945,347 noninstitutionalized adults resided in the surrounding area, increasing to 963,897 by 2019. Ten neighborhoods in the catchment area had no ADI score. Of the 539 neighborhoods included in the analysis, 108 had an ADI score <20 (least deprived), 325 had an ADI score of 21 to 80 (2nd to 4th quintile), and 106 had an ADI score >80 (most deprived). Overall, the UCC utilization rate increased from 64.6 visits/1,000 to 71.3 visits/1,000 after the opening of the FSED (χ*^2^*=968.73, *P*<0.001). Examination of the least deprived to the most deprived neighborhoods in the catchment area showed a decrease in visits to the UCC clinic per 1,000 people in the 1st and 2nd ADI quintiles and an increase in utilization in the 4th and 5th (most deprived) quintiles after the FSED opened. The majority of visits to the FSED came from the 5th quintile (most deprived) neighborhoods (120.2 visits per 1,000 people), followed by the neighborhoods in the ADI 4th quintile (32.5 visits per 1,000 people) and 3rd quintile (14.3 visits per 1,000 people).

**Table 4.** Population, Census Block Groups, and Visits per 1,000 People to the Urgent Care Clinic and Freestanding Emergency Department Overall and by Area Deprivation Index

				Urgent Care Clinic			
Variable	Adult Population, 2015	Adult Population, 2019	Census Block Groups	Before,^a^ Visits/Visits per 1,000 People	After,^a^ Visits/Visits per 1,000 People	*P* Value^b^	Freestanding Emergency Department, Visits/Visits per 1,000 People
Catchment area	945,347	963,897	549	61,122 / 64.6	68,733 / 71.3	<0.001	25,444 / 26.4
Area Deprivation Index quintile^c^							
1st	220,349	236,837	108	3,301 / 15.0	2,894 / 12.2	<0.001	883 / 3.7
2nd	227,422	236,385	112	5,300 / 23.3	4,949 / 20.9	<0.001	1,634 / 6.9
3rd	186,541	186,653	109	7,904 / 42.4	8,229 / 44.1	0.013	2,663 / 14.3
4th	158,143	157,355	104	12,859 / 81.3	14,764 / 93.8	<0.001	5,117 / 32.5
5th	131,965	126,051	106	31,758 / 240.6	37,897 / 300.6	<0.001	15,147 / 120.2

^a^Before, 26 months before the opening of the freestanding emergency department (October 1, 2015, to November 15, 2017); After, 28 months after the opening of the freestanding emergency department (November 16, 2017, to March 1, 2020).

^b^Chi-square test of independence.

^c^No Area Deprivation Index score was available for 10 neighborhoods in the catchment area.

Note: *P*<0.05 indicates statistical significance.

## DISCUSSION

Our study has 2 important findings. First, opening an FSED in a healthcare-access-deprived, low socioeconomic status area resulted in the treatment of significantly more Black patients. Second, opening a triaged-based FSED did not significantly impact the total number of visits to an adjacent UCC but resulted in a reduction of emergent visits to the UCC. Furthermore, opening an FSED provided the population with medical services during the hours the UCC was closed.

Overall, we found that opening an FSED in a poor, healthcare-resource-scarce area resulted in significantly more patients from deprived neighborhoods being treated at the FSED and UCC. In our study, the majority of patients presenting to the FSED and UCC were from the most deprived neighborhoods based on the ADI. This increase is likely related to the growing patient population in the Baton Rouge metropolitan area and surrounding regions.^27,28^ The increase in visits could also be attributable in part to the requirement that FSEDs in Louisiana have to receive ambulances,^4^ providing an additional stop for emergency medical services to stabilize critical patients in route to a hospital-based ED. As a hospital-affiliated FSED, the FMOL FSED provides acute medical care to all patients.

The opening of the FSED had no impact on the total number of nonemergent visits at the UCC, but emergent visits at the UCC decreased. This decrease is likely largely related to the triage system. Ho and colleagues noted that because of overlap in services provided by EDs and UCCs, patients may pay more for services in the ED that could have been delivered at a lower cost at a UCC.^1^ Our study demonstrates that a triage system is beneficial in identifying patients’ needs and addressing them with the appropriate level of care. Because of our triage system, patients without emergent needs are not required to pay for ED-level treatment and are treated at a less expensive level of care. However, during the hours that the UCC is closed, all patients are triaged to the FSED. The increase in the proportion of nonemergent visits and ESI level 2 (less urgent) visits to the FSED during UCC closed hours highlights the need for nonemergency services at all hours in resource-scarce neighborhoods.

### Limitations

To our knowledge, this study is the first to examine the impact of an FSED next to an established UCC in a low socioeconomic status area. However, several limitations must be considered. First, Baton Rouge has hospitals and UCCs in the city that are not within the FMOL Health System and are not located near the FMOL FSED service area. We were unable to examine the impact that opening the FSED had on those entities. Our study examines a single FSED and a single UCC using a unique triage system, potentially limiting the generalizability of our results. In addition, other factors may have impacted FSED utilization during the study period. For example, during this study period, natural disasters such as a flood and hurricanes occurred that may have had impacts on FSED utilization. In addition, Louisiana expanded Medicaid in 2016, and Medicaid expansion also significantly impacted FSED and UCC utilization rates. Finally, we used the ADI as a proxy for neighborhood poverty because we were not able to obtain the socioeconomic information for individual patients.

## CONCLUSION

Our study demonstrates that opening an FSED next to an established UCC in an impoverished area provides greater options for the treatment of economically disadvantaged citizens, allowing more nonemergent and emergent visits to be treated. Future work is necessary to have a broader understanding of the impact of an FSED on the city overall; however, our work demonstrates the feasibility and necessity of managing the medical needs of socially disadvantaged citizens through the combination of UCCs and FSEDs.

## Notes

*This article meets the Accreditation Council for Graduate Medical Education and the American Board of Medical Specialties Maintenance of Certification competencies for Patient Care and Medical Knowledge.*
